# Early-onset Alzheimer’s disease with depression as the first symptom: a case report with literature review

**DOI:** 10.3389/fpsyt.2023.1192562

**Published:** 2023-04-27

**Authors:** Meichen Liu, Xueting Xie, Jinghui Xie, Shiyun Tian, Xuemei Du, Hongbo Feng, Huimin Zhang

**Affiliations:** ^1^Department of Neurology, The First Affiliated Hospital, Dalian Medical University, Dalian, China; ^2^Department of Nuclear Medicine, The First Affiliated Hospital, Dalian Medical University, Dalian, China; ^3^Department of Radiology, The First Affiliated Hospital, Dalian Medical University, Dalian, China

**Keywords:** depression, Alzheimer’s disease, positron emission tomography, magnetic resonance imaging, neuroimaging, case report

## Abstract

**Background:**

Alzheimer’s disease is a common neurodegenerative disease, and patients with early-onset Alzheimer’s disease (onset age < 65 years) often have atypical symptoms, which are easily misdiagnosed and missed. Multimodality neuroimaging has become an important diagnostic and follow-up method for AD with its non-invasive and quantitative advantages.

**Case presentation:**

We report a case of a 59-year-old female with a diagnosis of depression at the age of 50 after a 46-year-old onset and a 9-year follow-up observation, who developed cognitive dysfunction manifested by memory loss and disorientation at the age of 53, and eventually developed dementia. Combined with neuropsychological scales (MMSE and MOCA scores decreased year by year and finally reached the dementia criteria) and the application of multimodal imaging. MRI showed that the hippocampus atrophied year by year and the cerebral cortex was extensively atrophied. 18F-FDG PET image showed hypometabolism in right parietal lobes, bilateral frontal lobes, bilateral joint parieto-temporal areas, and bilateral posterior cingulate glucose metabolism. The 18F-AV45 PET image showed the diagnosis of early-onset Alzheimer’s disease was confirmed by the presence of Aβ deposits in the cerebral cortex.

**Conclusion:**

Early-onset Alzheimer’s disease, which starts with depression, often has atypical symptoms and is prone to misdiagnosis. The combination of neuropsychological scales and neuroimaging examinations are good screening tools that can better assist in the early diagnosis of Alzheimer’s disease.

**Graphical Abstract:**
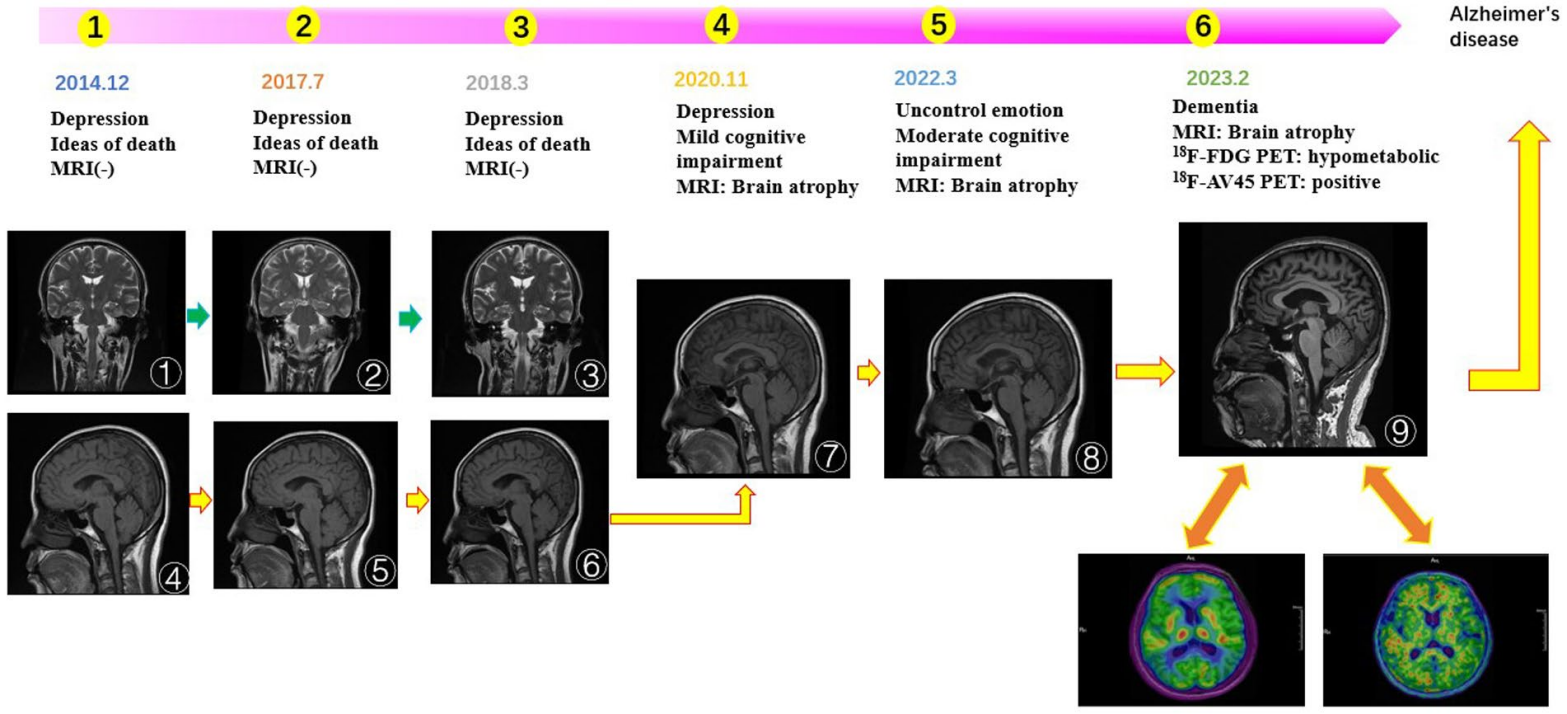

## 1. Introduction

Alzheimer’s disease is a progressive neurodegenerative disease and the most common type of dementia, accounting for about 60 to 80% (1). This disease is commonly seen in elderly people over 65 years of age, with memory impairment and psychobehavioral abnormalities as the main clinical manifestations (2). Magnetic resonance imaging of the head shows atrophy of the frontal, temporal, and parietal cortex, increased width of the temporal horn of the lateral ventricle, and atrophy of the internal olfactory cortex, amygdala, and hippocampus (3). The main pathological manifestations of AD are characterized by plaques formed by extracellular amyloid (A*β*) aggregates and neurofibrillary tangles (NFTs) formed by abnormal intracellular tau protein phosphorylation aggregates (4).

In recent years, studies have found that the pathological changes in AD begin 15 years before the onset of clinical symptoms and that irreversible damage to the nervous system has usually occurred by the time the diagnosis is made (5). Mild cognitive impairment is often considered to be a precursor stage of AD, and epidemiological surveys show that approximately 10 to 15% of patients with MCI develop AD every year, which is significantly higher than the 1 to 2% conversion rate of AD in normal aging (6). Recently, there has been a gradual increase in reports of early onset AD (EOAD) starting under the age of 65, which is estimated to account for about 5 to 10% of AD (7). However, the diagnosis of EOAD is more difficult, often takes longer from onset to diagnosis, and requires the use of more imaging and laboratory tests to find evidence of pathological changes to support the AD diagnosis. Therefore, early recognition of AD is particularly important for the diagnosis and treatment of diseases.

So far, with the development of molecular imaging, differential diagnosis can be made from multiple perspectives, such as structure, function, and pathology. In 2018, the National Institute on Aging and Alzheimer’s Disease (NIA-AA) proposed a biomarker-based “ATN” research framework (7). (A) refers to a positive brain Aβ-PET image; (T) refers to a positive Tau-PET image; and (N) is a brain ^18^F-FDG PET with hypometabolism and/or brain atrophy on MRI. Not only can ^18^F-FDG PET imaging distinguish AD patients from normal elderly people, but it can also identify patients at risk of progression to AD from MCI patients, with sensitivities and specificities of 70 and 90%, respectively (8). In addition, ^18^F-FDG PET can be used to identify APOE4 gene carriers. A study of 18F-FDG PET imaging in 209 APOE4 carriers and controls found that APOE4 gene carriers had age-independently decreased metabolism in the posterior cingulate gyrus and parietal lobe (9). The typical pattern of glucose metabolism in AD is bilateral temporoparietal hypometabolism, with progressive hypometabolism in the frontal lobe but without involvement of areas associated with sensation and movement (10).

Currently, research has shown that amyloid imaging is the best choice for the early diagnosis of AD. Amyloid PET can provide neuropathological information. As one of the main pathological markers of AD, the non-invasive, *in vivo* detection of amyloid plaques has a very high sensitivity and specificity (11). A meta-analysis assessing amyloid PET to predict conversion of mild cognitive impairment to Alzheimer’s disease showed a sensitivity of 93% (95% CI 73.1–99.9) and a specificity of 56% (47.2–65.8) (12). Thus, appropriate criteria for the use of amyloid PET have been proposed in the literature, identifying the following patient groups that are most likely to benefit from the test: patients with uncertainly mild cognitive impairment in the clinical setting; patients with dementia syndromes suggestive of Alzheimer’s disease but within atypical or suspected mixed etiologies; and patients with early-onset progressive cognitive decline (13). Although it cannot distinguish between amyloidosis diseases, and even some elderly people are positive, a negative test can rule out AD (14).

However, patients with AD not only have altered molecular metabolism but also changes in brain structure and function. Fortunately, PET/MRI allows simultaneous acquisition of molecular imaging as well as structural and functional imaging data, reducing operative time and improving patient compliance. Thanks to the development of multimodal MRI, resting-state functional MRI can be used to examine functional connectivity to look for specific patterns of functional activation, which can differentiate AD from disorders with similar clinical symptoms (15). Several studies combined functional connectivity with a structural approach, suggesting that structural abnormalities may be responsible for abnormal functional connectivity in diseases (16, 17).

As we all know, the default mode network (DMN) participates in cognitive processes such as situational memory, introspection, and emotion. In addition, the DMN is divided into the anterior DMN (medial prefrontal cortex, dorsal prefrontal cortex, anterior cingulate gyrus, lateral temporal lobe, etc.) and the posterior DMN (ventral prefrontal cortex, posterior cingulate gyrus, inferior parietal lobule, angular gyrus, hippocampus, medial temporal lobe, etc.) (18). A study of resting FDG-PET/fMRI data was conducted to investigate changes in the DMN subsystem in AD patients, functional connectivity and glucose metabolism were significantly reduced in patients with post-DMN, and the strength of functional connectivity in pDMN patients was positively correlated with the overall cognitive status of AD patients (19). Through PET and rs-fMRI studies, it was found that A*β* is first deposited in the posterior cingulate gyrus, precuneus, and temporoparietal regions in MCI, reducing functional connectivity between these regions and other brains and thus affecting the functional integrity of the DMN (20). The frontoparietal network (FPN) plays an important role in the regulation of behavior and the development of complex plans (21). The executive control network (ECN) plays an important role in the management and control of high-level cognition such as attention, decision-making, and working memory (22). A study using MCI patients as subjects found that the left FPN was positively correlated with the connectivity of the DMN, ECN, and memory storage in MCI (23). All in all, the above studies suggest that the impairment of specific functional networks underlies the clinical symptoms of AD, and that diminished regulatory effects lead to an overall impairment of cognitive function.

Depression has the potential to develop into dementia, and the potential relationship requires more research on the pathogenesis of depression and dementia. Current research suggests that chronic low-grade inflammation is associated with neurodegenerative diseases and psychiatric disorders, which may be a key point in connecting the pathogenesis of both (24). Because depression is associated with reduced levels of 5-hydroxytryptamine *in vivo*, most research has focused on the field of the serotonin pathways, but people are increasingly focusing on the KYN pathway, which produces various bioactive compounds related to inflammation, the immune system, the nervous system, and psychiatric disorders (25, 26). Moreover, several studies have shown that the tryptophan-kynurenine (TRP-KYN) metabolic pathway is closely associated with the immune regulation of depression and Alzheimer’s disease (27–29). Indeed, tryptophan is not only the main raw material for the synthesis of 5-hydroxytryptamine, it can also be degraded into several other neuroactive compounds, mainly kynurenine, kynurenic acid, which has neuroprotective effects, and quinine phosphate, which can cause degeneration of hippocampal neurons.

Recently, it has been reported that low levels of TRP are associated with AD, and supplementation with tryptophan can enhance cognitive function (30). Besides, another study suggests that KYNA has an antidepressant-like effect and that KYNA analogues are potential anti dementia agents (31). A previous study indicated that KP metabolites were highly correlated with biomarkers of neurofibrillary tau pathology, neuroinflammation, and neurodegeneration, suggesting that dysregulated KP metabolism may play a role in the pathogenesis of AD (32). In addition, a meta-analysis showed that TRP, KYN, and KYNA levels were decreased, KYN/TRP ratios were increased, and KYNA/KYN, KYNA/QA, and KYNA/3-HK ratios were decreased in patients with major depression (33). There are many other studies that have demonstrated the importance of KYN system activation in depression (34, 35). Thus, dysregulation of the TRP-KYN pathway, leading to increasing or decreasing neuroactive metabolites, is closely associated with Alzheimer’s disease and depression (29, 36).

Now a case of a patient initially diagnosed with depression and finally diagnosed with early-onset Alzheimer’s disease after 9 years of follow-up observation is reported below.

## 2. Case description

A 59-year-old female patient in junior high school began illness in 2010. After a family conflict, she felt sadness, decreased interest, self-blame, worry, concern, always thinking badly, panic, chest tightness, overall pain, dry mouth, loss of appetite, abdominal distention, and belching. Accompanied by sleep disturbances, manifested by difficulty in falling asleep, easy waking up at night, dreaminess, and difficulty in falling asleep after waking. Unfortunately, she did not see a doctor or get any remedy at that time.

In December 2014, she came to our hospital and felt that being alive was meaningless, what’s more, she had suicidal thoughts and was lazy to say and move. Besides, she did not like to do housework and even did not wash her face. Blood routine, biochemical liver function, kidney function, electrolytes, thyroid function, urinary routine, hepatitis virus, syphilis, and HIV were normal. No abnormality was found on the MRI examination of the head. The patient had a previous history of psoriasis spanning more than 20 years. Allergy to painkillers and fish. She denied a history of hypertension, diabetes, cerebrovascular disease, and heart disease. No history of surgery or trauma. No history of attraction or alcohol consumption. Denied the history of hereditary disease, infectious disease. We have a diagnosis of “depression,” and antidepressant treatment with duloxetine hydrochloride. Initially, outpatient visits were conducted every 2 weeks, but then they were changed to outpatient visits every 4 weeks. The above symptoms were completely relieved, and life functions were restored.

In July 2017, there was another negative life event; she experienced depression, decreased interest, reduced volitional behavior, and the idea of suicide reappeared. Memory loss occurs for the first time, such as forgetting to put salt in cooking. MMSE scale: 23 points. MOCA scale: 20 points. No abnormalities on head MRI.

In March 2018, the patient was followed up with a partial resolution of emotional symptoms and no improvement in memory. The MMSE scale and the MOCA scale were administered and there was no significant change in the scores. No abnormalities on head.

In November 2020, the follow-up continued, there was a delay in thinking, irritability, and being unable to control her temper. MMSE 23 points and MOCA 17 points. A head MRI showed signs of brain atrophy. Diagnosis: 1. depression 2. cognitive dysfunction given duloxetine hydrochloride antidepressant combined with memantine for improving cognitive function.

In March 2022, the patient had significantly worse memory loss, slowed thinking, and was unable to do housework. MMSE 19 points, MOCA 12 points. The MRI showed signs of brain atrophy. Following up with her family members, the patient has not taken her medication regularly since 2021. We readjusted the dosage of the medication and advised her to take it on time.

In February 2023, the patient had worsened memory loss due to recent events, disorientation, and personality changes, such as forgetting what she ate for breakfast and moving around the house in the wrong direction. More seriously, the patient used to be frugal but now often buys things she does not need. MMSE 14 points. MOCA 12 points. PET/MRI showed brain atrophy in the bilateral hippocampus, right amygdala, bilateral frontal, temporal, occipital, and right parietal lobes. DWI and SWI did not show any abnormal signals. The 18F-FDG image showed decreased glucose metabolism in the right parietal lobe, bilateral partial lobe, bilateral frontal lobe, bilateral parietotemporal joint region, and bilateral partial posterior cingulate gyrus. The 18F-AV45 PET image showed Aβ deposition was present in the cerebral cortex. Finally, we diagnose Alzheimer’s disease.

Adjusting the medication venlafaxine 150 mg to improve mood, gradually add memantine 5 mg to 10 mg, then 5 mg in the morning and 10 mg at night. During a follow-up visit 2 weeks after discharge, the patient’s family reported that the patient had increased daytime sleep when taking 15 mg, so it was adjusted to 15 mg at night to alleviate daytime sleepiness.

## 3. Diagnostic

**Figure 1:**
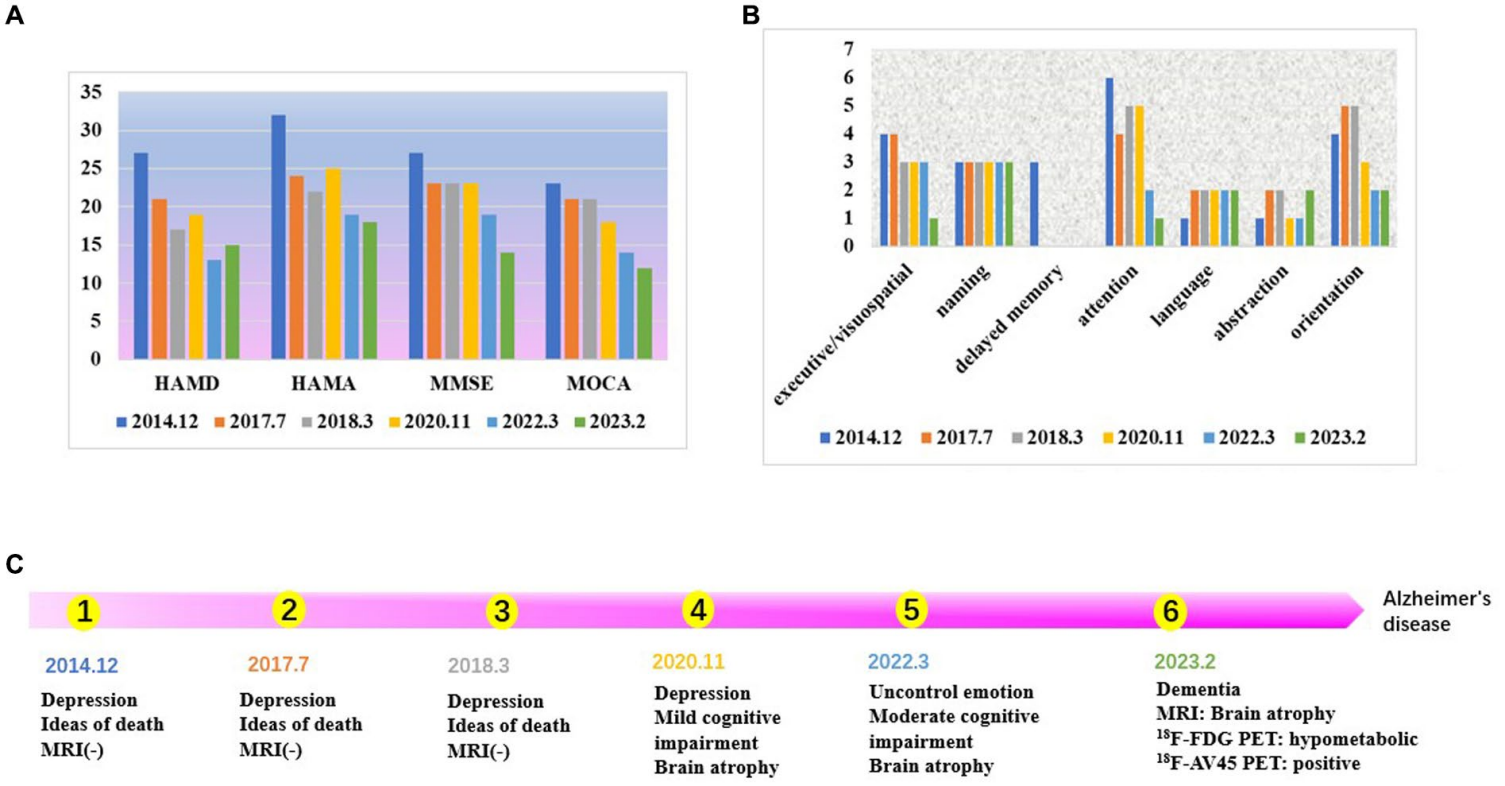
**(A)** The score of neuropsychological scale. The patient’s depression and anxiety scores, are decreasing. The scores for cognitive, dysfunction are increasing year by year. **(B)** The score of each cognitive domain in MOCA. Visuospatial/executive function, delayed memory, attention, and orientation are declined year to year. **(C)** Time line.

The improvement in the patient’s depression and anxiety symptoms after treatment can be seen in Figure 1A. However, symptoms of cognitive dysfunction progressively worsened over time. Signs of brain atrophy appear on imaging tests 10 years after the onset of the disease. Figure 1B shows a year-to-year decline in visuospatial/executive function, delayed memory, attention, and orientation in. Studies have found that delayed memory impairment in Alzheimer’s disease patients is a characteristic hallmark of the disease and is primarily related to hippocampal function (37). It was found that hippocampal atrophy is a valid predictor of whether MCI converts to AD, and the more hippocampal atrophy in MCI patients, the higher the rate of conversion to AD (37).

**Figure 2:**
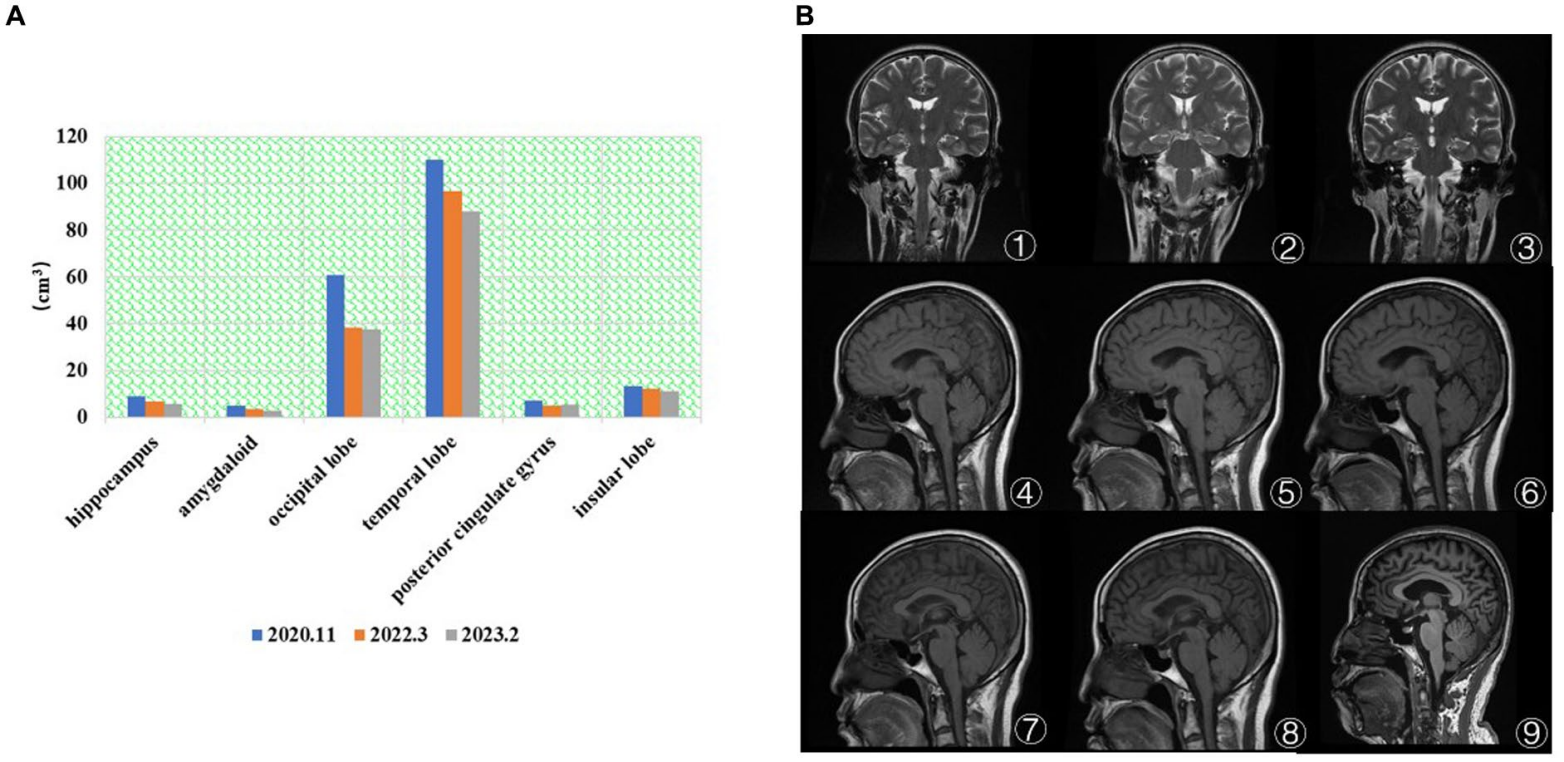
**(A)** The brain volume changes in regions of interest. The patient’s hippocampal, amygdala, occipital, temporal, and insula brain area volumes have gradually decreased over the last 3 years. **(B)** MRI results (①–⑨). ① 2014.12 Coronal T2. ② 2017.7 Coronal T2. ③ 2018.3 Coronal T2. ④ 2014.12 Coronal T1. ⑤ 2017.7 Coronal T1. ⑥ 2018.3 Coronal T1. ⑦ 2020.11 Coronal T2. ⑧ 2022.3 Coronal T1. ⑨ 2023.2 Coronal T1. ①–⑥ No significant atrophy in the hippocampal region. ⑦–⑨ The degree of brain atrophy gradually increased.

As shown in Figure 2A, the patient’s hippocampal, amygdala, occipital, temporal, and insula brain area volumes have gradually decreased over the last 3 years, consistent with the literature (38). There was an overall fluctuating decrease in the volume of brain regions in the posterior cingulate gyrus. In Figure 2B①–③, the visual analysis of the coronal scan showed no significant atrophy in the hippocampal region of the patient. As shown in Figure 2B④–⑥, the patient did not show any significant signs of brain atrophy on MRI follow-up from 2014.12 to 2018.3. As shown in Figure 2B⑦–⑨, the patient’s MRI from 2020.3 to 2023.2 showed signs of brain atrophy, and the degree of atrophy gradually increased.

After 3 years of treatment, the patient’s depressive symptoms were partially improved. Dramatically, during the treatment follow-up, it was found that the patient was indifferent, her family complained of her being unable to find home, her memory was reduced, she was unable to cook, and chronic cognitive symptoms such as memory and executive function gradually came to the fore. While emotional symptoms improved during treatment, cognitive symptoms did not. We performed an MRI examination and found no abnormalities in the blood routine, glutathione, glutamic aminotransferase, creatinine, and immune-related indicators. Apart from cerebrovascular diseases, there was no evidence for dementia symptoms in nutritional and metabolic diseases. There were no visual hallucinations, no RBD, and no basis for the dementia with Lewy body. Moreover, there is insufficient evidence for slow onset and gradual progression, such as in CJD and autoimmune brain disease. There is no history of fever and infection, and there is no evidence of infectious diseases such as encephalitis. The current evidence is more supportive of neurodegenerative diseases, and the possibility of frontotemporal dementia needs to be considered. However, the clinical manifestations of frontotemporal dementia are progressive impairments of behavior, executive function, and language, while memory impairment is often not significant. Only symptoms and signs alone are sometimes difficult to identify, so imaging studies are performed.

**Figure 3:**
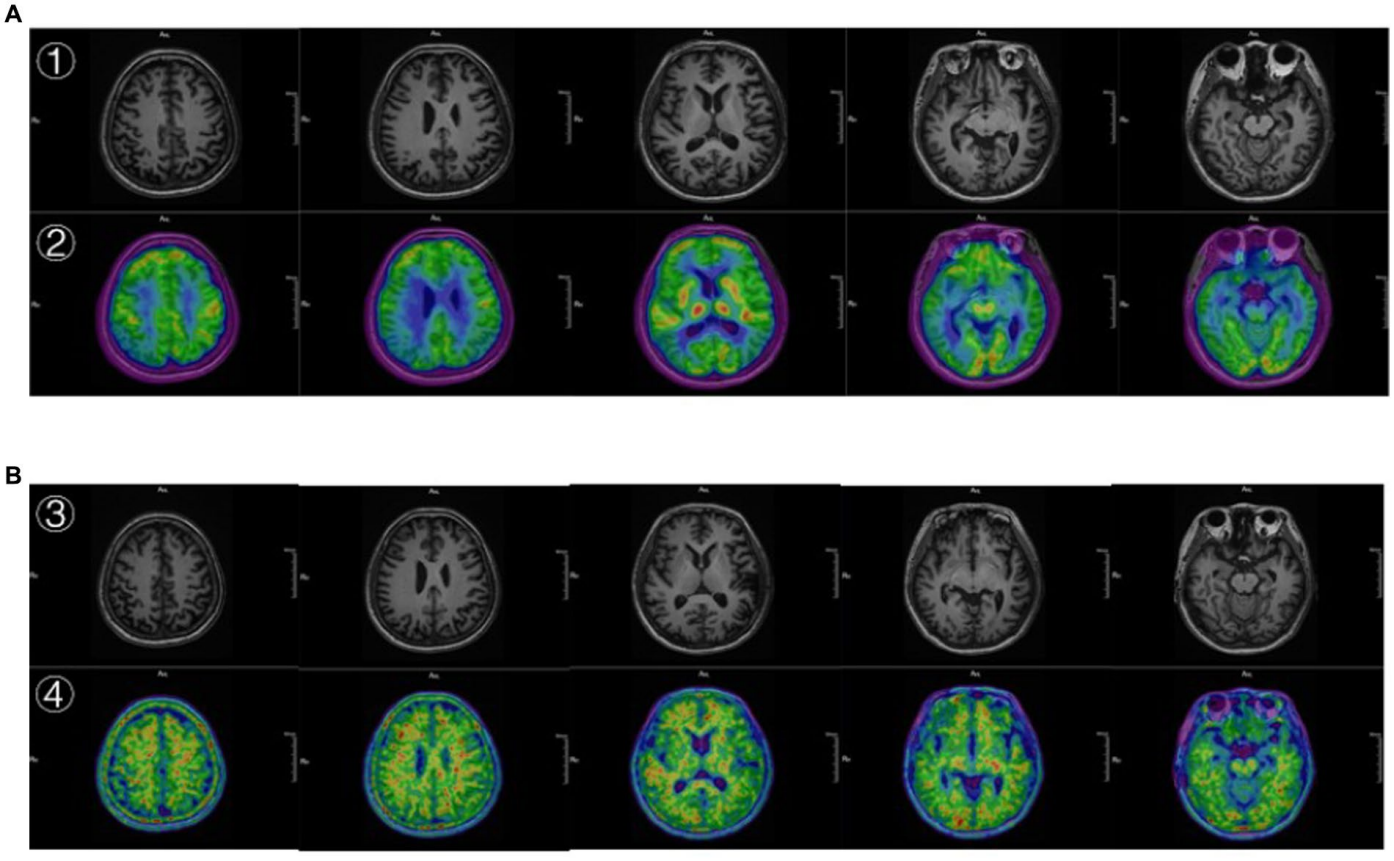
**(A)** ① The 3D T1 MRI. ② the image ^18^F-FDG PET fused with MRI. ② Showed hypometabolism in the parietal, temporal, and posterior cingulate gyrus. **(B)** ③ The 3D T1 MRI. ④ The image ^18^F-AV45 PET fused with MRI. ④ Showed significant cortical A*β* deposition.

The MRI structural imaging showed hippocampal and cortical atrophy, 18F-FDG imaging suggested hypometabolism in the parietal, temporal, and posterior cingulate gyrus (Figure 3A), and 18F-AV45 imaging suggested significant cortical A*β* deposition (Figure 3B), which is consistent with the imaging manifestations of Alzheimer’s disease (39). In contrast, patients with frontotemporal dementia show hypometabolism in the frontal and prefrontal lobes, and as the disease progresses, the involved area expands to the parietal and temporal lobes, subcortical basal ganglia area, and the thalamus, and has negative or mildly elevated A*β* imaging deposits, so it can be excluded (40). The areas of hypometabolism in dementia with Lewy body mostly involve the occipital lobe (41).

## 4. Discussion

The patient, who started with depressive symptoms at the age of 46, developed memory impairment after 7 years of follow-up. Then, imaging changes were found 10 years later, and a diagnosis of EOAD was made 13 years later. It is reported that the prevalence of EOAD is about 24.2/100000, and the annual incidence rate is about 6.3/100000 (42). Depression is one of the most common psychiatric disorders in AD patients, and it is also a risk factor for the onset of AD. Between 20 and 40% of AD patients experience depression, which often occurs early in the disease. Patients with early-onset AD are more likely to develop depression than those with late-onset AD due to significant changes in lifestyle, roles, and responsibilities, as well as poor social adaptability (43). More and more studies have shown that depression not only accelerates the rate of cognitive decline and affects the quality of life but also increases mortality and suicide rates in AD patients (44). Seriously, patients with Alzheimer’s disease whose depressive symptoms are the first to occur are prone to misdiagnosis.

In another systematic review study, which pooled 57 studies with a total of 20,892 MCI patients, the prevalence of depression was 32% (45). Consequently, the relationship between depression and cognitive impairment is close and complex, involving the interaction of biological and psychosocial factors. Current research suggests that AD and depression share some common pathological features, such as neuroinflammation, reduced synapse numbers, increased glucocorticoid levels, decreased BDNF levels, and decreased levels of monoamine neurotransmitters (46). In a study of 69 cases of mild cognitive impairment with 18F-FDG PET/CT patients who progressed to dementia showed hypometabolism in the parietal, temporal, and precuneus lobes (47, 48). Moreover, patients with depression have hypometabolism in the frontal lobe and anterior cingulate gyrus. On the contrary, patients who developed dementia without depression had increased metabolism in the frontal, parietal, and precuneus lobes. Hence, the metabolic patterns of depression and dementia overlap and differ from each other.

It has been proposed that the key to the treatment of AD lies in screening patients who may convert to AD during the MCI phase (49) and intervening early so as to delay the progression of the disease and even block its progression to AD (50). Current research suggests that Aβ deposition can be used as one of the detection indicators for the progression from MCI to AD. In a study of 184 subjects with 18F-AV45, it was found that about 76% of AD patients exhibited positive amyloid plaques, and for patients with MCI, about 38% of MCI patients showed 18F-AV45 retention in the brain, while 14% of the normal controls also showed Aβ deposition (51). In addition, there is a significant correlation between cortical Aβ deposition and age, as well as the ApoE4 gene. Notably, Aβ imaging saturates at an early stage of the disease, making Aβ poorly correlated with disease severity and unable to track progression (52).

Luckily, glucose metabolism was imaging compensates for the deficiencies. Specifically, in patients with mild AD, it was found that decreased metabolism in the unilateral temporal, parietal, or temporoparietal lobes, with the left side being more affected than the right side, and the proportion of temporal lobe involvement was slightly higher than that of the parietal lobe, but the frontal lobe was not affected. As the disease progresses, the frontal cortex is involved in the middle and late stages, and typical imaging appears, bilateral frontal, parietal, and temporal lobe metabolism is significantly reduced in a symmetrical manner (53). Not only that, the degree and range of metabolic reduction are positively correlated with the severity of clinical manifestations, but the metabolism of the striatum, thalamus, main sensory motor cortex, visual cortex, and cerebellum is relatively preserved. This pattern is consistent with the study by Caroli et al. (39). In summary, the frontal lobe can be used to distinguish early and mid to late stages of AD. Some studies suggest that AD is more prone to decreased metabolism in the posterior cingulate gyrus than FTD, while FTD is more prone to accumulate regions such as the basal ganglia than AD (50, 54). These findings are helpful in the diagnosis. Hence, if the FDG-PET and Aβ-PET are combined, it can contribute to improving the accuracy of AD and reducing misdiagnosis.

The CNS inflammatory response is mainly mediated by microglia and astrocytes. So the hyperactivation of microglia continuously releases inflammatory factors that promote neuroinflammation (55), and the activated microglia that mediate the neuroinflammation are associated with Aβ、Tau protein (56). Furthermore, during the onset of major depressive disorder, there is also a correlation between neuroinflammation in the brain and the activation of microglia (57). At present, the most common target for PET imaging of neuroinflammation is the 18KDa transporter protein (TSPO). A study used TSPO-PET to study AD and showed that the medial temporal lobe, temporoparietal lobe, and cingulate cortex are the main brain areas where radioactive uptake is distributed (58). Another study performed PET scans on MDD patients and found increased TSPO VT in the PFC, ACC, and insular lobe, and the larger the TSPO VT in the ACC, the higher the severity of depression (57). This finding demonstrates that the neuroinflammatory process is related to microglia activation, which provides a supportive environment for developing new treatment methods by reducing microglia activation. The combination of TSPO-PET and the TRP/KYN pathway, which is an important pathway for neuroinflammation, may provide a reference for further elucidating the pathogenesis of depression and AD.

## 5. Limitations and future directions

However, our research has some limitations. First of all, the patient did not undergo genetic testing and cerebrospinal fluid testing in this case for personal reasons and was unable to analyze the genotype of the EOAD. We are trying to further communicate with patients with a view to achieving genetic testing at a future follow-up. Secondly, due to the limitations of our unit, tau protein PET and TSPO-PET could not be performed to demonstrate the imaging features of Alzheimer’s disease in a multi-dimensional way. Thirdly, the case is rather homogeneous, and it is hoped that in the future, prospective trials on depression with cognitive impairment can be carried out, combining the TRP-KYN pathway with multimodal imaging to find new biological markers to facilitate early diagnosis and detect new therapeutic targets.

## 6. Conclusion

In general, we report a case of early-onset Alzheimer’s disease with depression as the first symptom, which was followed for up to 9 years. Through a literature review, the potential pathogenesis of depression and Alzheimer’s disease was analyzed in terms of molecular imaging, functional imaging, inflammation, and metabolism. It is hoped to draw clinicians’ attention to patients with atypical and early-onset AD, so as to better identify and intervene early in AD, as well as to discover the potential new targets for pharmacological treatment of both diseases, which is of great significance for the treatment of depression and AD in the future.

## Data availability statement

The original contributions presented in the study are included in the article/supplementary material, further inquiries can be directed to the corresponding author.

## Ethics statement

Ethical review and approval was not required for the study on human participants in accordance with the local legislation and institutional requirements. The patients/participants provided their written informed consent to participate in this study. Written informed consent was obtained from the individual(s) for the publication of any potentially identifiable images or data included in this article.

## Author contributions

ML, XX, and HZ were responsible for assessing the condition and patient follow-up. JX, ST, XD, and HF collected the image data. All authors contributed to the article and approved the submitted version.

## Conflict of interest

The authors declare that the research was conducted in the absence of any commercial or financial relationships that could be construed as a potential conflict of interest.

## Publisher’s note

All claims expressed in this article are solely those of the authors and do not necessarily represent those of their affiliated organizations, or those of the publisher, the editors and the reviewers. Any product that may be evaluated in this article, or claim that may be made by its manufacturer, is not guaranteed or endorsed by the publisher.
